# BCL10 Deficiency Presenting as Severe Combined Immunodeficiency Escaping Newborn Screening

**DOI:** 10.1007/s10875-023-01646-w

**Published:** 2023-12-30

**Authors:** Sarah Salou, Simon Voelkl, Baerbel Keller, Stephan Ehl, Nora Naumann-Bartsch

**Affiliations:** 1 Center for Chronic Immunodeficiency (CCI), Medical Center University of Freiburg, Faculty of Medicine, University of Freiburg, Freiburg, Germany; 2 Center for Pediatrics and Adolescent Medicine, Medical Center, Faculty of Medicine, University of Freiburg, Freiburg, Germany; 3 Department of Internal Medicine 5, Hematology and Oncology, University Hospital Erlangen, Erlangen, Germany; 4 Department of Rheumatology and Clinical Immunology, Medical Center - University of Freiburg, Faculty of Medicine, University of Freiburg, Freiburg, Germany; 5 Clinic for Children and Adolescents, Department of Hematology and Oncology, University Hospital Erlangen, Erlangen, Germany

To the Editor:

We report on a 5-month-old male infant with complete B-cell lymphoma/leukemia 10 (BCL10) deficiency presenting with fatal severe combined immunodeficiency (SCID) characterized by systemic adenovirus infection and *Pneumocystis jirovecii* pneumonia.

The boy was born at term as the first child of healthy nonconsanguineous parents from the Balkan countries. Newborn screening was normal including TREC (T cell receptor excision circles) based screening for SCID. Within the first 3 months of age, the infant developed properly, and vaccinations were performed as scheduled including two oral doses of the Rotavirus vaccine. No adverse effects were observed after immunization. However, at the age of 3 months, he developed chronic diarrhea and failed to thrive.

At the age of 5 months, the infant presented to a local hospital due to high fever, coughing, tachydyspnea, and dehydration. He rapidly developed hypoxemic respiratory failure requiring supplemental oxygen therapy. X-ray of the chest revealed pneumonic infiltrates, so he was started on antibiotic treatment. Furthermore, intravenous immunoglobulins were administered and oral therapy with Aspirin was started since atypical Kawasaki disease was also suspected due to persisting febrile episodes. Multiplex PCR assays from the patient’s throat swab and stool sample were positive for adenovirus (ADV). After initial stabilization, the patient again developed a high fever, high C-reactive protein (CrP) levels, and progression of pneumonic infiltrates associated with increasing oxygen demand. Despite the escalation of the antibiotic regimen and high-flow oxygen therapy, the patient progressed to respiratory failure and required invasive ventilation. The patient had moderate leukocytopenia of 3260/µl with a normal proportion of lymphocytes. Immunoglobulin G level was low despite previous intravenous application, and immunoglobulin A and M levels were just above the detection limit. ADV load increased rapidly in the tracheal secretion and the peripheral blood (up to 1 × 10^8 copies/ml) and PCR from the patient’s airway secretion was also positive for *Pneumocystis jirovecii* (PCJ). The stool was negative for other pathogens. Despite treatment with cidofovir, high-dose cotrimoxazole, and additional immunoglobulins, the patient’s condition remained critical. The combination of failure to thrive due to very early onset of chronic diarrhea, prolonged systemic infection caused by ADV that was further aggravated by PCJ, as well as the laboratory finding of hypogammaglobulinemia in a child with normal TREC screening was highly suspicious for a SCID variant without T cell lymphopenia and prompted further diagnostics to characterize the patient’s immunophenotype.

Absolute T cell numbers (CD3^+^, CD4^+^, CD8^+^), B cells (CD19^+^), and NK cells (CD16^+^/CD56^+^) were within normal ranges (Fig. 1A) with 74% of CD4 + CD45RA + cells being recent thymic emigrants (CD31 +) (data not shown) and Vβ-analysis revealed a diverse T cell receptor (TCR) repertoire in CD4^+^ and CD8^+^ subsets (data not shown). However, despite the disseminated adenoviral infection, there was a very high rate of naïve T cells (CCR7^+^CD45RA^+^ CD3^+^) of 97.6% and thus nearly complete absence of memory T cells and low activated HLA-DR + T cell subsets (Fig. 1A). Furthermore, CD4^+^ T cells of the patient showed reduced or nearly absent proliferation after stimulation with PHA and anti-CD3 + / − anti-CD28, respectively (Fig. 1B). Defective T-cell activation was also demonstrated by reduced upregulation of the activation marker CD25^+^/CD69^+^ after stimulation of CD4^+^ T cells with PHA and antiCD3 + CD28 beads (Fig. 1C). Lack of FoxP3 expressing CD4^+^ T cells pointed out impaired differentiation of regulatory T cells (Fig. 1D). A defect in T-cell activation was suspected and further analysis showed missing IκBα degradation and p65 phosphorylation after stimulation in naïve CD4^+^ T cells with anti-CD3/CD28 and PMA (Fig. 1E). Furthermore, IκBα degradation and p65 phosphorylation were absent after stimulation of naïve B cells with anti-IgM but completely normal after stimulation with CD40L, pointing toward impaired signaling via the CARD11-BCL10-MALT1 complex.

**Fig. 1:**
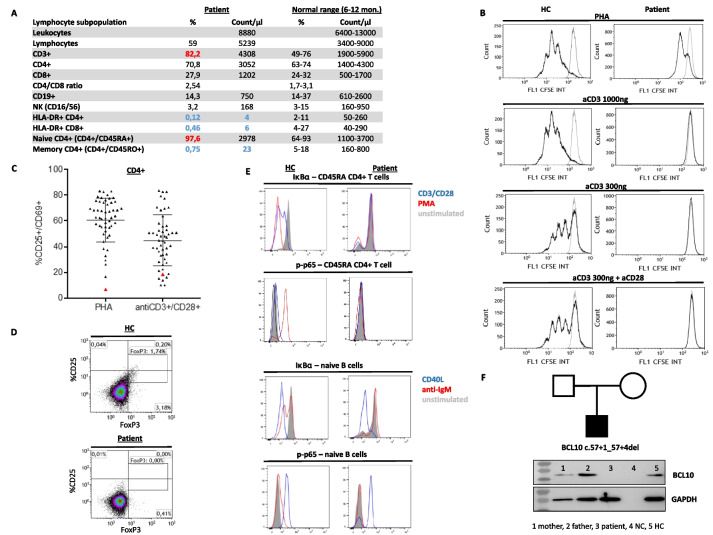
Immunophenotyping of the BCL10 deficient patient. **A** Lymphocyte phenotyping (standard values according to Shearer et al. JACI (2003) and Hulstaert et al. Clin Immunol Immunopathol (1994)). **B** Stimulation of CD4^+^ T cells with PHA and antiCD3 ± CD28 in the patient and a healthy control (HC). **C** Scatter plot visualizes T-cell activation represented as upregulation of the activation marker CD25^+^/CD69^+^ after stimulation of CD4^+^ T cells with PHA and antiCD3 + CD28 beads in the patient (red triangle) compared to healthy controls (gray triangle, *n* = 50). **D** Staining for FoxP3 in CD4^+^ T cells of the patient and a healthy control (HC). **E** Analysis of IκBα degradation and p65 phosphorylation after stimulation of naive CD4^+^ T cells with anti-CD3/CD28 and PMA as well as after stimulation of naive IgMpos CD27neg CD19pos B cells with anti-IgM and CD40L. **F** Pedigree and protein expression of BCL10 in PBMC by immunoblot analysis (method as described in Garcia–Solis et al. [1]) of the patient, his parents, negative control (NC), and healthy control (HC)

Genetic testing for CARD11, MALT1, and BCL10 revealed a homozygous gene mutation in *BCL10* (IVS1 c.57 + 1_57 + 4del chr1: g.85741975_85741978del) that has not been previously described. To test whether this alteration leads to impaired splicing on the RNA level, RNA was isolated and analyzed from bone marrow–derived mononuclear cells and showed a homozygous insertion of 492 nucleotides of intron 1 (r.57 + 1_57 + 4delins57 + 5_57 + 496) that is predicted to cause a premature stop codon. Immunoblot analysis from PBMC confirmed complete BCL10 deficiency in the patient (Fig. 1F).

An emergency preparation of allogenic adenovirus-specific T cells as bridging therapy to allogenic hematopoietic stem cell transplantation was immediately initiated. However, despite intensive treatment, the patient deteriorated rapidly with progressive respiratory failure. Eventually, he died due to multiorgan failure and disseminated cerebral infarction causing brain edema. The autopsy confirmed massive pneumonia, hepatitis, and necrotizing encephalitis caused by fulminant systemic ADV infection.

Human BCL10 deficiency (OMIM #616098) is an inborn error of immunity (IE) that was first reported in 2014 and is classified in the literature as combined immunodeficiency (CID) [2]. The gene *BCL10* encodes for B cell CLL/lymphoma 10 (BCL10), a member of the CARD11-BCL10-MALT1 (CBM) complex. After cell stimulation, BCL10 forms a complex with caspase recruitment domain-containing (CARD) family adaptors (CARD9 and CARD11) and mucosa-associated lymphoid tissue lymphoma translocation protein 1 (MALT1) which induces activation of NF-κB in lymphoid cells and therefore is essential in immune cell activation [2].

To date, there have been four published cases of infants with BCL10 deficiency [1–4]. All patients initially presented at or within the first year of life with bronchopulmonary and gastrointestinal viral and bacterial infections. However, in three cases, the clinical course extended beyond 1 up to 3 years of life, and while one infant had mild BCGitis, the other two did not show signature opportunistic infections of SCID [1–3]. In contrast, our patient presented with disseminated adenovirus and PCJ infection leading to death at 6 months of age, the typical clinical course of SCID patients. Al-Tamemi et al. [4] described a similar course in a 7-month-old female infant with BCL10 loss-of-function mutation who had a history of recurrent severe infections and eventually died. These two patients add to the report of Greil et al. [5] describing a child with CARD11 deficiency presented with PCJ within the first 6 months of life. A further patient with MALT1 deficiency has been reported as SCID but had a less severe presentation [6]. All of these patients had severely impaired T-cell proliferation.

In our opinion, the question of whether to call these phenotypes SCID or CID is more than semantic. For most pediatricians, the diagnosis of SCID is directly connected to an immunological emergency requiring immediate specialist attention in a center for stem cell transplantation. This is not the case for CID, in which a wide range of phenotypes makes transplantation seem a less urgent matter. We strongly suggest maintaining the concept of urgency associated with the SCID diagnosis also in the days of newborn screening for severe T cell lymphopenia. SCID continues to exist, we need to teach our fellows how to recognize it and to label it properly so the necessary urgency on the way to life-saving therapy is maintained. The present report adds BCL-10 deficiency to other defects of the CBM complex and other diseases of T cell activation such as ORAI1 or STIM1 deficiency that can present as SCID in babies with normal SCID screening. In our hands, a high percentage of naïve T cells despite significant viral infections and reduced T cell proliferation has served as a useful first diagnostic clue to these diseases.

## Data Availability

Not applicable.

## Abbreviations

ADV: Adenovirus
BCL10: B-cell lymphoma/leukemia 10
CBM complex: CARD11-BCL10-MALT1 complex
CARD: Caspase recruitment domain-containing protein
CID: Combined immunodeficiency
CrP: C-reactive protein
MALT1: Mucosa-associated lymphoid tissue lymphoma translocation protein 1
PBMC: Peripheral blood mononuclear cells
PCJ: *Pneumocystis jirovecii*
SCID: Severe combined immunodeficiency
TCR: T cell receptor
TREC: T cell receptor excision circles
